# Flow cytometric analysis of the immune cell subsets in adult hemophagocytic lymphohistiocytosis

**DOI:** 10.3389/fimmu.2025.1678233

**Published:** 2025-11-28

**Authors:** Yao Qin, Jianying Zhou, Pengpeng Liu, Yang Dai, Yun Tang, Xushu Zhong, Xinchuan Chen, Huanling Zhu, Ting Liu

**Affiliations:** Department of Hematology, Institute of Hematology, West China Hospital of Sichuan University, Chengdu, Sichuan, China

**Keywords:** hemophagocytic lymphohistiocytosis, immune cell subsets, Epstein-Barr virus, PD-1 inhibitor, flow cytometry

## Abstract

**Purpose:**

Hemophagocytic lymphohistiocytosis (HLH) is a hyperinflammatory syndrome characterized by the dysfunction of cytotoxic T cells (CTLs) and natural killer (NK) cells. The status of these immune cells during persistent inflammation remains unclear. This study aimed to investigate the immune cell subsets, the exhaustion and activation status, and the cytotoxic functions of the CTLs and NK cells in adult HLH. Furthermore, the features of immune cells in patients with Epstein–Barr virus-associated HLH (EBV-HLH) before and after treatment with programmed death-1 (PD-1) inhibitors were also analyzed.

**Methods:**

Flow cytometry was utilized to evaluate the immune cell features in 75 patients with adult HLH.

**Results:**

This study observed skewed immune cell subsets, along with substantial exhaustion and activation in patients with adult HLH. Significant decreases were noted in NK, natural killer T (NKT), regulatory T cells (Tregs), CD4^+^ central memory T (T_CM_), CD8^+^ terminal effector memory T (T_EMRA_), and CD56^dim^CD16^+^ NK cells, while increases were found in the CD56^bright^CD16^−^ and CD56^dim^CD16^−^ NK subsets. The exhaustion markers (PD-1, TIGIT, and PD-L1) were elevated in CD8^+^ T cells and NK cells, and the activation markers (CD69, HLA-DR, and CD163) were higher in monocytes. An impaired IFN-γ production and a reduced degranulation function were also detected in CD8^+^ T cells and NK cells. In EBV-HLH patients treated with PD-1 inhibitors, we observed an increase in cytotoxic CD8^+^ T cells and CD56^dim^CD16^+^ NK cells and a decrease in CD56^bright^CD16^−^ NK cells.

**Conclusion:**

These findings suggest impairments in cytotoxic immune cells along with a widespread immune exhaustion and an aberrant activation in adult HLH. The results also indicate potential differentiation defects in CD8^+^ T cells and NK cells. Treatment with a PD-1 inhibitor may contribute to partial functional recovery and support viral clearance in EBV-HLH, which warrant further investigation.

## Introduction

Hemophagocytic lymphohistiocytosis (HLH) is a rare but life-threatening hyperinflammatory condition caused by the dysfunction of cytotoxic T cells (CTLs) and natural killer (NK) cells. This syndrome is characterized by a pronounced immune activation of macrophages and T cells (1). The hallmark clinical features include prolonged fever, hepatosplenomegaly, and pancytopenia. Typical laboratory findings encompass elevated levels of triglycerides, ferritin, and soluble interleukin 2 (IL-2) receptor, alongside reduced levels of fibrinogen and a diminished NK cell activity (2). Pathologically, HLH is distinguished by the presence of hemophagocytosis in the bone marrow, the spleen, or the lymph nodes (3).

HLH can be categorized into primary HLH (pHLH) and secondary HLH (sHLH). The most common form of pHLH, familial HLH, predominantly affects children, whereas sHLH is more prevalent in adults. The common triggers for sHLH include infections, malignancies, and autoimmune diseases (4). The pathogenesis of sHLH is multifactorial, with one underlying cause often being predominant. This includes endogenous factors such as genetic defects that affect cytotoxic function and background inflammation and exogenous factors such as immunosuppression and infectious triggers. These factors interact to eventually reach a threshold at which inflammation becomes uncontrolled, leading to fulminant HLH (5). Insights into HLH have primarily emerged from studies of familial HLH, where genetic defects compromise the cytotoxic functions of NK cells and CTLs, resulting in an impaired ability to eliminate infected or malignant cells (6). This dysfunction can lead to the activation of antigen-specific T cells, which in turn causes the hyperactivation of both T cells and macrophages, culminating in a cytokine storm (7). The resultant cytokine response syndrome could cause tissue damage, multiple organ dysfunction, and even death (8). Despite these insights, the precise pathogenesis of adult HLH remains elusive, particularly as most cases do not involve homozygous or compound HLH-related mutations. Further investigation into the status and the function of immune cells in patients with adult sHLH is warranted.

Epstein–Barr virus-associated hemophagocytic lymphohistiocytosis (EBV-HLH) is a fatal type of HLH triggered by EBV infection (4, 9) that is particularly prevalent in East Asian populations and has a worse prognosis compared with the other infection-associated HLH subtypes (10, 11), with a 1-year mortality rate reaching 78% in adults and adolescents and an average survival period of only 3.5 months (12). Currently, there is no standardized treatment for adult EBV-HLH. The most commonly used regimen, the HLH94/2004 protocol (3), primarily manages the inflammatory response, but does not effectively control the EBV infection, leading to a high relapse rate (13). Allogeneic hematopoietic stem cell transplantation remains the only curative option (14, 15). Our previous work has demonstrated the safety and efficacy of the programmed death-1 (PD-1) inhibitor nivolumab in the treatment of patients with relapsed and refractory EBV-HLH, showing potential to restore normal T-cell function against EBV infection and resulting in clinical improvements (16).

This study investigated the differences in the immune cell subsets, the exhaustion and activation status, and the cytotoxic functions of the CTLs and NK cells in patients with adult HLH. In addition, we explored the alterations in EBV-HLH patients before and after treatment with a PD-1 inhibitor to better understand their impact on disease progression and outcomes.

## Methods

### Ethics approval

The study was approved by the Committee of West China Hospital of Sichuan University (HX-IRB 2020-887). Individual informed written consent was obtained from each patient and participant.

### Patients and healthy subjects

The diagnosis of HLH was made according to the criteria of the HLH-2004 protocol (3). A panel of 42 HLH-related genetic mutations was analyzed using next-generation sequencing (NGS) (**Supplementary Table S1**). A total of 75 adult patients were enrolled and diagnosed in West China Hospital between September 2020 and June 2023, and 10 healthy subjects were recruited as controls. All patients were treated based on the HLH-2004 protocol, and 12 of them with EBV-HLH were treated initially with the same regimen as “cooling therapy” to reduce the clinical symptoms and then with a PD-1 inhibitor (sintilimab; Innovent Biologics, Suzhou, China), administered intravenously at a fixed dose of 100 mg every month. Peripheral blood samples for immune profiling were collected at baseline (prior to the first sintilimab infusion) and post-treatment for response assessment (days 30, 60, and 90).

### Flow cytometry analysis

Peripheral blood (3–5 ml) was collected from HLH patients and healthy controls (HCs) using lithium heparin as an anticoagulant. All samples were processed and analyzed in whole blood format within 6 h after collection. The sample was lysed using Red Blood Cell Lysis Buffer (C3702; Beyotime, Shanghai, China). Cell viability was assessed with trypan blue exclusion using a Countstar^®^ Automated Cell Counter, which was consistently >90% at the time of staining. For surface marker staining, the cells were incubated with specified anti-human monoclonal antibodies (mAbs) for 20 min at room temperature, protected from light. After washing twice, the cells were resuspended in 500 μl 1× phosphate-buffered solution (pH 7.2–7.4) and analyzed on FACS Canto Flow Cytometry using FlowJo software (version 10.8). For intracellular cytokine staining, the cells were cultured in RPMI-1640 supplemented with 2% fetal bovine serum for 5 h at 37°C under 5% CO_2_ in the presence of stimulation cocktails from eBioscience [2:1,000 dilution of 500× cocktail (00-4975-93) for IFN-γ detection or 1:1,000 dilution of 500× cocktail (00-4970-93) for granzyme B and perforin]. After stimulation, the cells were stained with anti-human mAbs specific for surface markers, fixed with an IC Fixation Buffer (00-8222-49; eBioscience, San Diego, CA, USA) for 30 min, protected from light, and then the cells were permeabilized with buffer (00-8333; eBioscience) and stained with anti-human mAbs specific against intracellular molecules. Subsequently, the cells were washed, resuspended, and immediately analyzed on FACS Canto flow cytometry.

Flow cytometry analysis utilized the following gating strategy (**Supplementary Figure S1**). White blood cells (WBCs) were gated as CD45^+^ cells. Monocytes were identified by excluding lymphocytes and neutrophils and were then defined by CD14 expression. Lymphocytes were categorized into B lymphocytes and CD3^+^/CD3^−^ T lymphocytes. CD3^+^ T cells were gated and subdivided into the CD4^+^CD8^−^ and CD4^−^CD8^+^ subsets. These were further divided into four subsets: naive T cells (T_naive_, CD45RA^+^CCR7^+^), central memory T cells (T_CM_, CD45RA^−^CCR7^+^), effector memory T cells (T_EM_, CD45RA^−^CCR7^−^), and terminal effector memory T cells (T_EMRA_, CD45RA^+^CCR7^−^). Regulatory T cells (Tregs) were defined as CD4^+^CD8^−^CD25^+^CD127^−^, while natural killer T (NKT) cells were identified by CD3^+^CD56^+^. Among the CD3^−^ cells, NK cells were identified as CD56^+^ and subdivided into the CD56^bright^CD16^−^ and CD56^dim^CD16^+^ subsets. Dendritic cells (DCs) were defined as HLA-DR^+^Lin^−^ cells, excluding the CD3^+^ T/B lymphocytes, monocytes, and NK cells (**Supplementary Figure 1A**). The inhibitory receptor markers (PD-1, TIGIT, and PD-L1) were detected on T cells, NK cells, NKT cells, monocytes, and DCs. Cells in blue represent isotype controls (**Supplementary Figure S1B**). Activation markers (CD69, HLA-DR, and CD163) were assessed on T cells, NK cells, and monocytes (**Supplementary Figure S1C**), while intracellular IFN-γ, perforin, and granzyme B were quantified in CD4^+^ T cells, CD8^+^ T cells, and NK cells (**Supplementary Figure S1D**).

### NK cell cytotoxicity and degranulation assay

The cytotoxicity of NK cells was assessed using a cytotoxicity assay against K562 cells. Peripheral blood mononuclear cells (PBMCs) were isolated via density gradient centrifugation and co-cultured with green fluorescent protein (GFP)-transfected K562 cells at a 10:1 effector-to-target ratio. Specifically, the CD107a-PE antibody (2 μl) (555801; BD, Franklin Lakes, NJ, USA) and monensin (6 μg/ml) (554724; GolgiStop, BD Biosciences, Franklin Lakes, NJ, USA) were added in at the start of incubation at 37°C under 5% CO_2_ for 4 h. Thereafter, the cells were stained with mAbs specific for NK cell surface markers and 7-AAD (17501; AAT Bioquest, Pleasanton, CA, USA). The positive controls consisted of NK cells stimulated with a phorbol 12-myristate 13-acetate and ionomycin cocktail (00-4970-93; eBioscience) and then co-cultured with K562 target cells, whereas the negative controls were unstimulated NK cells maintained without target cells. The cytotoxic activity of NK cells was determined based on the expression of CD107a and the percentage of 7-AAD^+^ K562 cells.

### Statistical analysis

SPSS version 25.0 was used for statistical analysis. All data are presented as median and range. Normality of data distribution was assessed using the Shapiro–Wilk test. Based on the distribution, either the Student’s *t*-test (for normally distributed data) or the Mann–Whitney *U* test (for non-normally distributed data) was applied for comparisons between two groups. The *p*-values were analyzed using a two-sided analysis, and a *p*-value less than 0.05 was considered statistically significant.

## Results

### Clinical characteristics of HLH patients

A total of 75 patients were enrolled in the study (24 men and 51 women; median age = 32 years, range = 14–83 years). Of these, 29 (38.70%) patients had HLH associated with infections, including 26 with EBV infection (34.7%), two with cytomegalovirus (CMV) infection (2.6%), and one with tuberculosis infection (1.3%). Malignancy-associated HLH was identified in 38 (50.70%) patients, including 21 (28.00%) lymphomas comprising 13 with NK/T cells, eight with B-cell subsets, and 17 (22.7%) cases of NK cell leukemia (NKL). Autoimmune disease-associated HLH (macrophage activation syndrome, MAS) was diagnosed in two patients (2.70%), while the triggers remained unclear in six patients (8%). Among all patients, 17 (22.67%) were found to have heterozygous mutations identified using a panel of 42 HLH-related genes through NGS. The characteristics of all enrolled patients are summarized in **Table 1**. A total of 10 healthy individuals (five men and five women), with a median age of 37 years (26–55 years), were enrolled as controls (HCs).

**Table 1:** Disease classification and clinical characteristics of the patients.

Characteristic	All HLH patients (*n* = 75)/percentage^a^	EBV-HLH patients (*n* = 26)/percentage^b^
Disease classification, *n* (%)		
EBV-HLH	26 (34.7)	26 (100)
Lym-HLH	21 (28)	–
NKL-HLH	17 (22.7)	–
MAS	2 (2.7)	–
Other infection-HLH^c^	3 (4.0)	–
Unknown^d^	6 (8.0)	–
Age (years)		
Median (range)	32 (14–83)	25.5 (14–60)
Gender, *n* (%)		
Women	51 (68)	13 (50)
Men	24 (32)	13 (50)
Presentation, *n* (%)		
Fever	75 (100)	26 (100)
Pancytopenia	62 (82.7)	20 (76.9)
Hepatosplenomegaly	64 (85.3)	22 (84.6)
Elevated ALT/AST	59 (78.7)	24 (92.3)
Hyperferritin	71 (94.7)	25 (96.2)
Elevated sIL2R	56 (74.7)	22 (84.6)
Hypertriglyceridemia	34 (45.3)	13 (50.0)
Hypofibrinogenemia	39 (52.0)	13 (50.0)
Elevated EBV-DNA	60 (80.0)	26 (100)

Pancytopenia: affecting at least two lineages in the peripheral blood.

*HLH*, hemophagocytic lymphohistiocytosis; *EBV-HLH*, Epstein–Barr virus-associated HLH; *Lym-HLH*, lymphoma-associated HLH; *NKL-HLH*, natural killer cell leukemia-associated HLH; *MAS*, macrophage activation syndrome; *ALT*, alanine aminotransferase; *AST*, aspartate aminotransferase; *sIL-2R*, soluble interleukin 2 receptor.

a Clinical characteristics of all HLH patients.

b Clinical characteristics of EBV-HLH patients.

c Cytomegalovirus and tuberculosis-associated HLH.

d Primary disease is not clear.

### Immune cell composition in HLH patients

**Figure 1:**
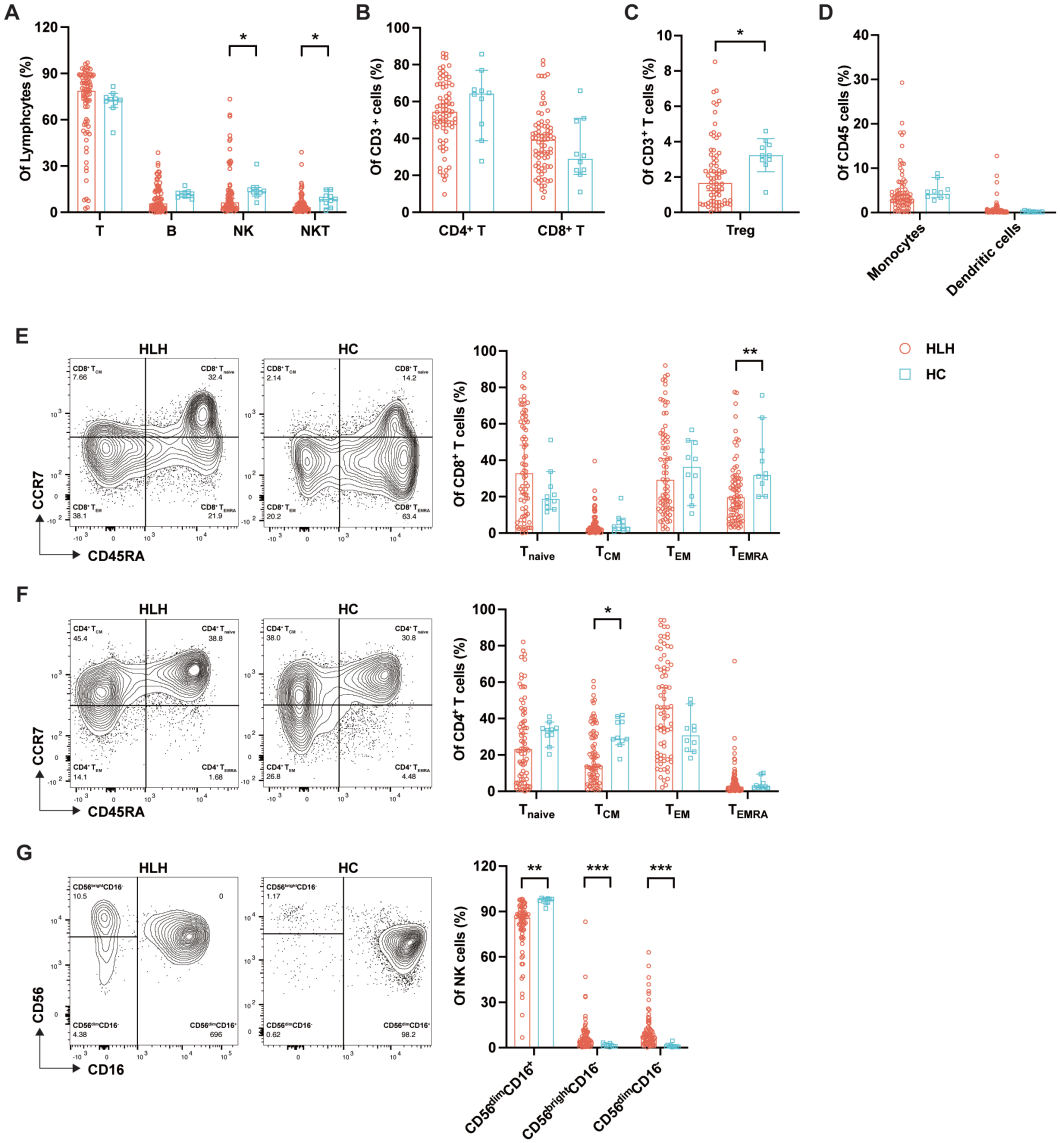
Analysis of the lymphocyte subsets and immunophenotypes in patients with hemophagocytic lymphohistiocytosis (HLH). Circulating lymphocytes from patients newly diagnosed with HLH and from healthy controls (HCs) were analyzed using flow cytometry. **(A)** Percentages of T cells, B cells, natural killer (NK) cells, and natural killer T (NKT) cells among lymphocytes in HLH patients and HCs. **(B)** Percentages of CD4^+^ and CD8^+^ T cells among the CD3^+^ T cells in HLH patients and HCs. **(C)** Percentages of regulatory T cells (Tregs) among the CD3^+^ T cells in HLH patients and HCs. **(D)** Percentages of monocytes and dendritic cells among the CD45^+^ cells in HLH patients and HCs. **(E)** Percentages of the CD8^+^ T-cell subtypes (T_naive_, T_CM_, T_EM_, and T_EMRA_) in HLH patients and HCs. **(F)** Percentages of the CD4^+^ T-cell subtypes (T_naive_, T_CM_, T_EM_, and T_EMRA_) in HLH patients and HCs. **(G)** Percentages of the NK cell subsets (CD56^dim^CD16^+^, CD56^bright^CD16^−^, and CD56^dim^CD16^−^) in HLH patients and HCs. Data are presented as median with 95% CI. *0.01 < *p* < 0.05; **0.001 < *p* < 0.01; ***p < 0.001.

The constituent of immune cells differed between HLH patients and HCs (**Supplementary Table S2**). We found decreased proportions of NK and NKT cells in patients with HLH compared with the control group (NK = 6.48% *vs.* 13.61%, *p* = 0.016; NKT = 3.31% *vs.* 8.58%, *p* = 0.025) (**Figure 1A**). The proportions of CD3^+^, CD4^+^, and CD8^+^ T cells, which are important components of acquired immunity, did not differ between HLH patients and HCs (**Figure 1B**). However, Tregs were significantly decreased in HLH (1.66% *vs*. 3.22%, *p* = 0.011) (**Figure 1C**), which may contribute to the uncontrollable overactivated inflammation in HLH. In contrast, the monocytes and DCs did not show significant differences between HLH patients and controls (**Figure 1D**).

T cells exert their function of eliminating pathogens or malignancy by differentiating into antigen-specific effector cells from memory or naive cells. The analysis of the T-cell subtypes based on differentiation stage showed a significant decrease of the CD8^+^ T_EMRA_ proportion in HLH (19.73% *vs*. 31.90%, *p* = 0.006), and the CD8^+^ T_naive_ cells in patients with HLH were higher than that in the controls, although the difference was not statistically significant (*p* = 0.210) (**Figure 1E**). These findings indicate an abnormal CD8^+^ T-cell differentiation, which is characterized by a reduction in terminally differentiated effector cells and a relative accumulation of the less differentiated subsets, which may reflect impaired cytotoxic maturation. Simultaneously, in the CD4^+^ T-cell subsets, the percentage of T_CM_ cells was significantly lower (*p* = 0.017), while the percentage of T_EM_ cells was higher in patients with HLH than in the controls (*p* = 0.167) (**Figure 1F**). This indicates that there is persistent antigenic stimulation in patients with HLH, leading to the differentiation of T_CM_ into T_EM_ (17). In addition, there was also a trend toward a decrease in the proportion of CD4^+^ T_EMRA_ cells in patients with HLH (*p* = 0.629).

NK cells can be classified into different subsets based on the expression levels of CD56 and CD16, including CD56^dim^CD16^+^, CD56^bright^CD16^−^, and CD56^dim^CD16^−^. CD56^dim^CD16^+^ NK cells constitute >90% of peripheral NK cells and exhibit stronger natural cytotoxicity, while the CD56^bright^CD16^−^ subset is more cytokine-producing and is less cytotoxic (18). It is currently accepted that CD56^dim^CD16^+^ NK cells are differentiated from CD56^bright^CD16^−^ NK cells (19). In patients with HLH, the proportion of the cytotoxic CD56^dim^CD16^+^ NK cell subset was significantly lower than that in the controls (86.00% *vs.* 98.04%, *p* = 0.000). Simultaneously, the proportions of the CD56^bright^CD16^−^ (4.88% *vs.* 1.16%, *p* = 0.001) and CD56^dim^CD16^−^ (7.69% *vs.* 0.85%, *p* = 0.000) subsets were significantly higher in patients with HLH (**Figure 1G**), suggesting the immature and less cytotoxic phenotypes of NK cells in HLH.

### Exhaustion and activation of immune cells in HLH patients

**Figure 2:**
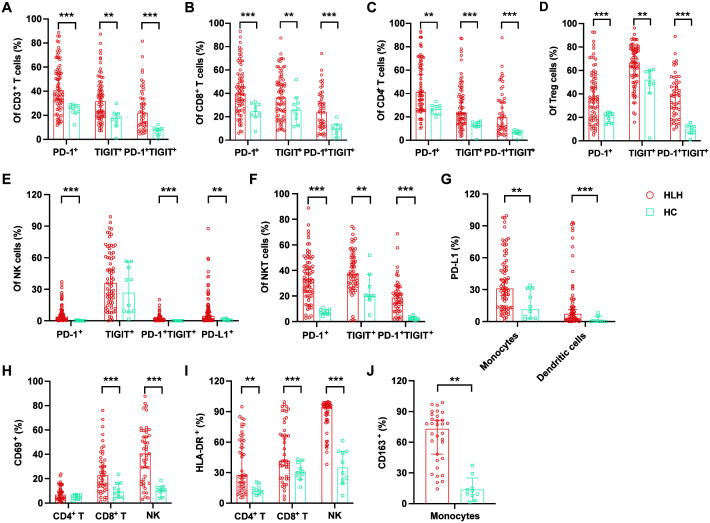
Immune exhaustion and activation in patients with hemophagocytic lymphohistiocytosis (HLH). **(A–D)** Proportions of PD-1^+^, TIGIT^+^, and PD-1^+^TIGIT^+^ cells among the CD3^+^, CD8^+^, and CD4^+^ T cells and the regulatory T cells (Tregs) in HLH patients and healthy controls (HCs). **(E, F)** Proportions of PD-1^+^, TIGIT^+^, PD-1^+^TIGIT^+^, and PD-L1^+^ cells among the natural killer (NK) and natural killer T (NKT) cells in HLH patients and HCs. **(G)** Proportions of PD-L1^+^ monocytes and dendritic cells in HLH patients and HCs. **(H)** CD69 expression levels on CD4^+^ T cells, CD8^+^ T cells, and NK cells. **(I)** HLA-DR expression levels on CD4^+^ T cells, CD8^+^ T cells, and NK cells. **(J)** CD163 expression levels on monocytes in HLH patients and HCs. Data are presented as median with 95% CI. *0.01 < *p* < 0.05; **0.001 < *p* < 0.01; ***p < 0.001.

A few studies have exhibited the exhausted features of peripheral immune cells in HLH, which result in hypofunctional effector T cells and may contribute to the pathogenesis of this syndrome. In this study, we observed increased proportions of PD-1^+^, TIGIT^+^, and PD-1/TIGIT co-positive cells in nearly all lymphocyte subsets in patients with HLH (**Table 2**), including CD3^+^, CD4^+^, and CD8^+^ T cells, Tregs, NK cells, and NKT cells (**Figures 2A–F**). In addition, patients with HLH exhibited a significantly increased expression of PD-L1 in NK cells, monocytes, and DCs (**Figures 2E, G**).

**Table 2:** Exhaustion and activation of immune cells in hemophagocytic lymphohistiocytosis (HLH) patients.

Cell subset	HLH (*n* = 75)	Control (*n* = 10)	*p*-value
Exhaustion			
CD3^+^ T cells, % (range)			
PD-1^+^	40.59 (11.63–88.73)	26.25 (12.00–29.19)	**0.001**
TIGIT^+^	31.63 (7.11–87.39)	17.40 (0.62–29.90)	**0.002**
PD-1^+^TIGIT^+^	22.00 (4.48–81.50)	7.02 (0.32–12.23)	**0.000**
CD4^+^ T cells, % (range)			
PD-1^+^	41.09 (10.27–93.49)	26.81 (19.80–33.38)	**0.004**
TIGIT^+^	23.01 (3.32–87.39)	12.95 (0.44–16.40)	**0.001**
PD-1^+^TIGIT^+^	19.70 (3.90–87.70)	6.33 (0.26–9.05)	**0.000**
CD8^+^ T cells, % (range)			
PD-1^+^	39.60 (5.99–92.96)	24.53 (11.46–32.80)	**0.000**
TIGIT^+^	36.48 (8.14–87.39)	25.85 (11.46–51.90)	**0.017**
PD-1^+^TIGIT^+^	24.30 (3.66–74.10)	9.53 (0.38–19.80)	**0.003**
Tregs, % (range)			
PD-1^+^	36.05 (4.90–92.68)	21.75 (14.10–24.40)	**0.000**
TIGIT^+^	66.69 (15.91–96.12)	52.00 (2.31–60.60)	**0.009**
PD-1^+^TIGIT^+^	38.60 (9.87–89.00)	9.49 (0.82–15.63)	**0.000**
NK cells, % (range)			
PD-1^+^	4.00 (0.18–37.13)	0.13 (0.01–0.89)	**0.000**
TIGIT^+^	36.05 (0.02–98.91)	26.80 (1.65–56.61)	0.206
PD-1^+^TIGIT^+^	1.63 (0.06–20.20)	0.03 (0.00–0.12)	**0.000**
PD-L1^+^	4.62 (0.08–87.61)	0.85 (0.15–2.58)	**0.003**
NKT cells, % (range)			
PD-1^+^	33.26 (2.69–88.89)	6.81 (4.05–10.50)	**0.000**
TIGIT^+^	37.07 (0.51–74.45)	20.17 (5.14–51.99)	**0.002**
PD-1^+^TIGIT^+^	18.70 (1.31–68.70)	2.48 (0.95–5.38)	**0.000**
Monocytes, % (range)			
PD-L1^+^	31.09 (0.85–99.41)	11.74 (2.18–34.07)	**0.009**
Dendritic cells, % (range)			
PD-L1^+^	7.14 (0.00–93.13)	0.26 (0.00–7.92)	**0.000**
Activation			
CD4^+^T cells, % (range)			
CD69^+^	6.84 (0.37–23.90)	5.22 (1.53–7.05)	0.221
HLA-DR^+^	27.65 (5.33–94.70)	12.05 (7.88–20.80)	**0.004**
CD8^+^T cells, % (range)			
CD69^+^	22.80 (1.32–76.00)	9.27 (3.59–23.60)	**0.000**
HLA-DR^+^	41.70 (3.64–99.60)	30.70 (15.80–43.20)	**0.000**
NK cells, % (range)			
CD69^+^	40.60 (3.06–87.60)	10.75 (3.94–18.60)	**0.000**
HLA-DR^+^	94.30 (38.80–99.90)	35.05 (7.46–61.40)	**0.000**
Monocytes, % (range)			
CD163^+^	73.05 (14.30–98.80)	10.98 (1.13–37.20)	**0.000**

*PD-1*, programmed death 1; *PD-L1*, programmed death-ligand 1; *TIGIT*, T-cell immunoglobulin and ITIM domain. The bold values indicate significant statistical significance (p-value < 0.05).

Hyperinflammation is a hallmark of HLH. Our results demonstrated a picture of a universally aberrant activation of immune cells, with increased expression of CD69 and HLA-DR on CD8^+^ T cells and NK cells, HLA-DR on CD4^+^ T cells, and CD163 on monocytes in patients with HLH compared with the controls (**Table 2**; **Figures 2H–J**).

### Cellular functional impairment in HLH patients

**Figure 3:**
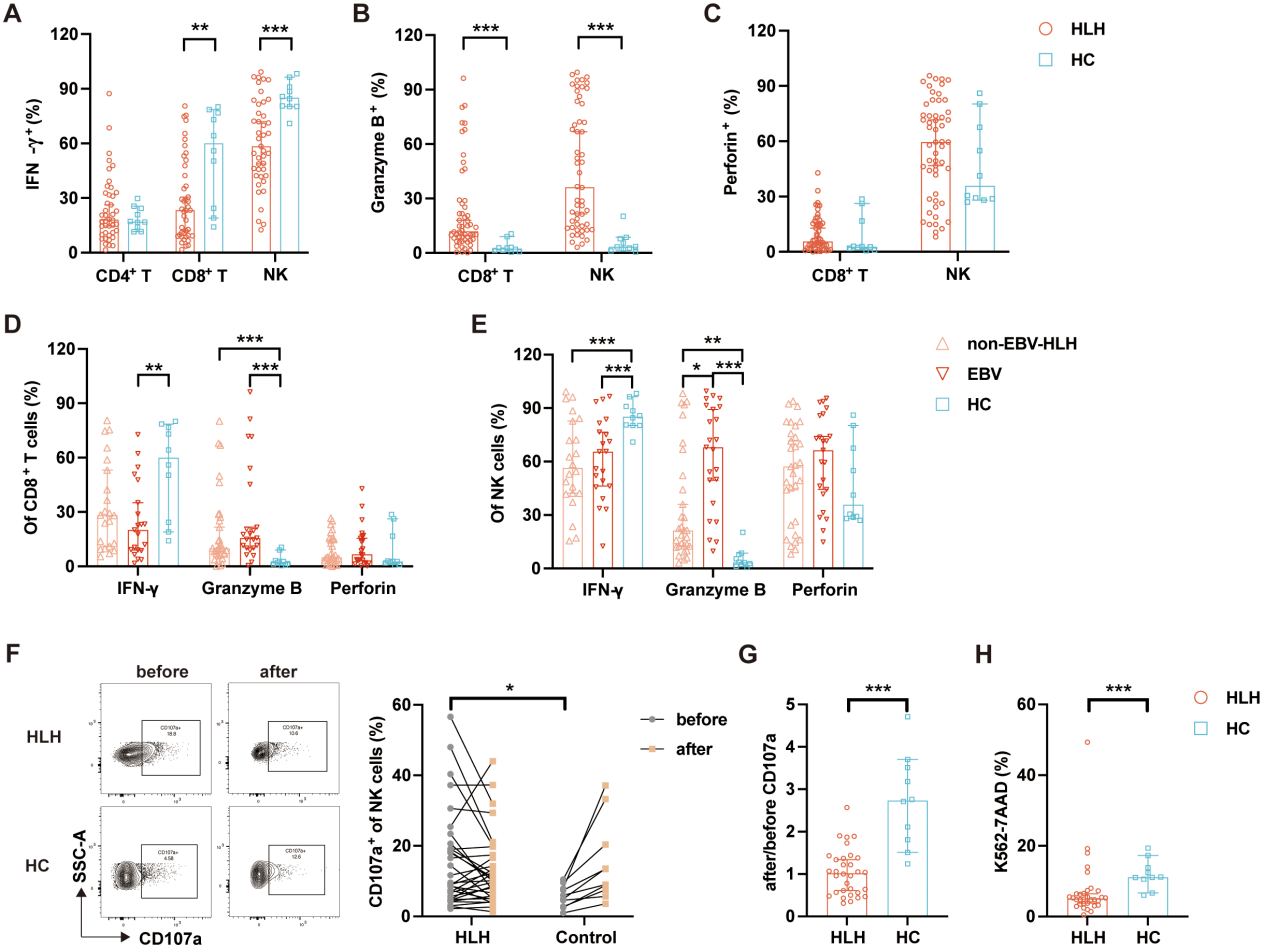
Cellular functional impairment in patients with hemophagocytic lymphohistiocytosis (HLH). **(A–C)** Proportions of IFN-γ^+^, granzyme B^+^, and perforin^+^ cells among the CD4^+^ T cells, CD8^+^ T cells, and natural killer (NK) cells in HLH patients and healthy controls (HCs). **(D, E)** Comparison of the proportions of IFN-γ^+^, granzyme B^+^, and perforin^+^ in CD8^+^ T cells and NK cells among the non-Epstein–Barr virus-associated HLH (non-EBV-HLH), EBV-HLH, and HC groups. **(F)** Representative flow plots and quantitative analysis of the expression of CD107a on NK cells before and after stimulation in HLH patients and HCs. **(G)** Ratio of CD107a^+^ NK cells after/before stimulation in HLH patients and HCs**. (H)** Proportion of K562-7AAD^+^ cells indicating NK cell cytotoxicity in HLH patients and HCs. Data are presented as median with 95% CI. *0.01 < *p* < 0.05; **0.001 < *p* < 0.01; ***p < 0.001.

Cytokines such as IFN-γ, granzyme B, and perforin are crucial in the pathogenesis of HLH, reflecting the functionality of T cells and NK cells. In this study, we assessed the expression levels of IFN-γ, granzyme B, and perforin in these cells (**Supplementary Table S3**). The results showed that the expression of IFN-γ in CD8^+^ T cells (23.39% *vs*. 60.09%, *p* = 0.008) and NK cells (58.40% *vs*. 85.20%, *p* = 0.000) was significantly lower in patients with HLH compared with the controls (**Figure 3A**). In addition, the levels of granzyme B in patients with HLH were significantly elevated in CD8^+^ T cells (11.98% *vs*. 2.41%, *p* = 0.000) and NK cells (36.28% *vs*. 3.43%, *p* = 0.000) (**Figure 3B**). Although the differences were not statistically significant, the levels of perforin trended higher in patients with HLH (**Figure 3C**). These results suggest a defect in granule release rather than production.

We further compared the functions of CD8^+^ T cells and NK cells among the patients with EBV-HLH and non-EBV-HLH. Patients with EBV-HLH showed a trend toward lower levels of IFN-γ in CD8^+^ T cells (28.23% *vs.* 20.10%, *p* = 0.494) and a higher granzyme B expression (9.94% *vs*. 15.60%, *p* = 0.418) (**Figure 3D**). Similarly, the expression levels of granzyme B in NK cells were markedly elevated in patients with EBV-HLH (21.40% *vs.* 68.10%, *p* = 0.014) (**Figure 3E**). These findings suggest more severe cytokine secretion defects in patients with EBV-HLH.

To further confirm the dysfunction in granule release, we assessed the expression of CD107a. Patients with HLH had a higher baseline expression of CD107a than the controls (*p* = 0.020) (**Supplementary Table S4**; **Figure 3F**). The ratio of CD107a expression increased significantly after stimulation in the control group (2.74-fold), while there was only a minimal 1.02-fold rise (*p* = 0.000) in patients with HLH (**Figure 3G**). In line with this, we observed significant reductions in NK cell cytotoxicity in patients with HLH compared with the controls (**Figure 3H**). These results suggest that the dysfunction in cytotoxic granule release directly contributes to the impaired cytotoxic activity of NK cells in patients with HLH, particularly in the EBV-HLH subgroup.

### Immune cell subset changes before and after PD-1 inhibitor treatment in EBV-HLH patients

**Figure 4:**
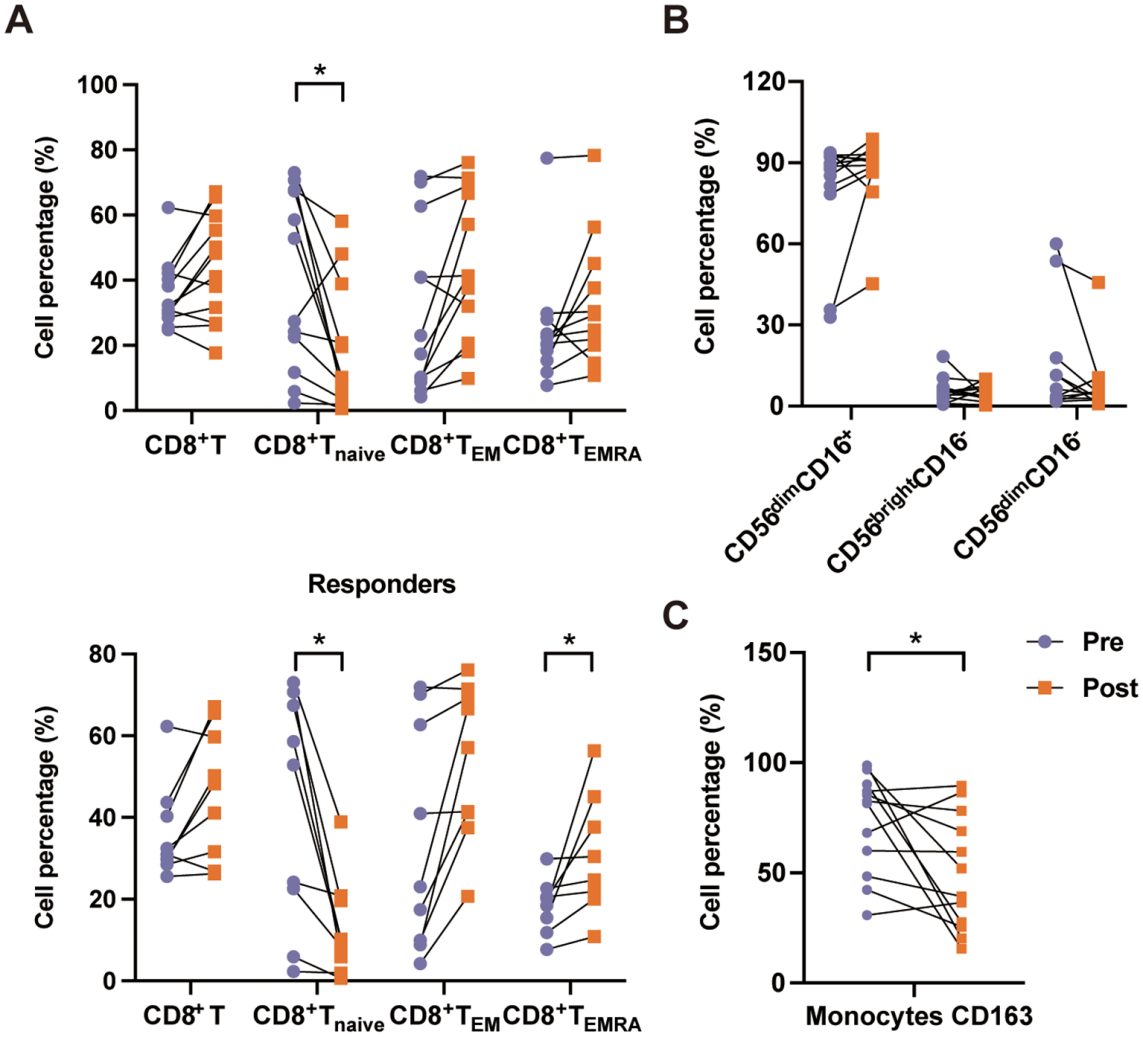
Immune cell subset changes before and after programmed death-1 (PD-1) inhibitor (PD-1i) treatment in patients with Epstein–Barr virus-associated hemophagocytic lymphohistiocytosis (EBV-HLH). **(A)** Changes in the percentages of the CD8^+^ T-cell subsets, including total CD8^+^ T cells, CD8^+^ T_naive_, CD8^+^ T_EM_, and CD8^+^ T_EMRA_, in HLH patients before (*Pre*) and after (*Post*) treatment. The *upper panel* shows the data for all EBV-HLH patients receiving PD-1i treatment, while the *lower panel* shows the data for responders. **(B)** Changes in the natural killer (NK) cell subsets, including CD56^dim^CD16^+^, CD56^bright^CD16^−^, and CD56^dim^CD16^−^, in HLH patients before and after treatment. **(C)** Percentages of CD163^+^ monocytes before and after treatment. Data are connected by *lines* to show paired comparisons for each patient. *0.01 < *p* < 0.05.

Previously, we used PD-1 inhibitors to treat patients with EBV-HLH and attained a >70% response rate (16). In this study, we examined the changes in the immune cell subsets before and after PD-1 inhibitor (sintilimab) treatment in 12 patients with EBV-HLH, of whom nine patients reached complete response and three patients showed non-response. After treatment, we observed an expansion of CD8^+^ T cells (31.65% *vs*. 44.65%, *p* = 0.219). More interestingly, PD-1 inhibition was associated with a shift in the T-cell differentiation profile: an increase in CD8^+^ T_EM_ cells (20.20% *vs.* 41.29%, *p* = 0.128) and T_EMRA_ cells (21.61% *vs.* 29.90%, *p* = 0.160), accompanied by a significant decrease in CD8^+^ T_naive_ cells (40.09% *vs.* 10.16%, *p* = 0.024). This trend was even more pronounced among responders, who showed a marked reduction in CD8^+^ T_naive_ cells (52.80% *vs*. 10.12%, *p* = 0.017) and an increase in CD8^+^ T_EMRA_ cells (20.20% *vs*. 30.40%, *p* = 0.048) (**Figure 4A**). However, no significant differences were observed in non-responders (**Supplementary Figure S2A**). A tendency of increase in CD56^dim^CD16^+^ NK cells (88.25% *vs.* 89.65%, *p* = 0.410) and decrease in CD56^bright^CD16^−^ NK cells (5.04% *vs.* 3.80%, *p* = 0.755) was observed after treatment, indicating the promotion of NK cell maturation (**Figure 4B**, **Supplementary Figure S2B**). Furthermore, a significant reduction in the expression of CD163 (82.05% *vs.* 45.70%, *p* = 0.033) indicated a downregulation of hyperinflammatory monocyte activation (**Figure 4C**, **Supplementary Figure S2C**).

## Discussion

HLH is a life-threatening hyperinflammatory disorder driven by persistent immune activation and an impaired cytotoxic function of CTL and NK cells. The results of this research revealed profound alterations in the composition and functional state of the immune cells in patients with adult HLH. Specifically, we observed significant decreases in NK cells, NKT cells, Tregs, CD4^+^ T_CM_ cells, CD8^+^ T_EMRA_ cells, and CD56^dim^CD16^+^ NK cells in patients with HLH, accompanied by an increase in T_naive_ cells and in the less differentiated CD56^bright^CD16^−^ and CD56^dim^CD16^−^ NK subsets. As CD8^+^ T_EMRA_ cells and CD56^dim^CD16^+^ NK cells are primary cytotoxic effectors (20, 21), their reduction likely contributes to the defective elimination of infected or malignant cells. Notably, although intracellular granzyme B was elevated in HLH-derived CD8^+^ T and NK cells, functional impairment in degranulation was evident, suggesting a defect in cytotoxic granule exocytosis rather than production.

A key finding is the disruption in NK cell maturation. The CD56^bright^CD16^−^ NK cell subset serves as a precursor to the CD56^dim^CD16^+^ NK cell subset within a linear differentiation model (18, 19). CD56^bright^CD16^−^ NK cells exhibit elevated levels of NKG2A, produce abundant cytokines, and possess a relatively low innate cytotoxicity (22). The increase in the proportion of CD56^bright^CD16^−^ NK cells in patients with HLH indicates potential disorders in NK cell differentiation. These are further supported by the markedly elevated granzyme B content within NK cells, particularly in EBV-HLH, indicating a “split anergy” phenotype wherein cells are metabolically active but are functionally retarded (23).

The observed paradox of an elevated intracellular granzyme B coupled with an impaired cytotoxicity may be primarily linked to a state of profound immune exhaustion of cytotoxic lymphocytes, which are recognized in the setting of persistent antigenic stimulation, such as in chronic viral infections and hyperinflammatory syndromes, including HLH (23, 24). It characterized by the high expression of inhibitory receptors such as PD-1 and TIGIT, as we have documented, which disrupt proximal T-cell receptor signaling and cytoskeletal reorganization. This signaling impairment results in the defective polarization of lytic granules toward the immune synapse and abrogated granule exocytosis, despite a preserved or an even upregulated production of cytotoxic mediators (7, 25). Recent studies have demonstrated that exhausted T and NK cells retain a high granzyme B content, but exhibit a severely blunted CD107a mobilization. Wang et al. (23) specifically identified this “armed but disabled” signature in sHLH, where the intracellular granzyme B accumulation coexists with poor cytotoxic function. Kelkar et al. (24) reported that the PD-1^+^CD8^+^ T cells in patients with HLH maintain high levels of cytotoxic molecules, but show a profound functional impairment. Such a dysregulation likely exacerbates the disease severity and is correlated with poor prognosis (17, 26).

We also found a significant reduction in Tregs, which may undermine the immune homeostasis and facilitate the hyperactivation of CD8^+^ T cells. Concurrently, an elevated and sustained expression of the early activation markers such as CD69 on T and NK cells was associated with impaired effector functions—a state of “aberrant activation.” Moreover, the increased HLA-DR expression across multiple lymphocyte subsets and the heightened CD163 on monocytes/macrophages align with prior studies (27–29) and our previous single-cell RNA sequencing data (16), underscoring the role of hyperactivated monocytes/macrophages in perpetuating inflammation (5).

Cellular immune exhaustion is another hallmark feature in the development of HLH. Our study found increased proportions of PD-1^+^, TIGIT^+^, and PD-1/TIGIT double-positive cells across CD3^+^, CD4^+^, and CD8^+^ T cells, Tregs, NK cells, and NKT cells, along with an elevated PD-L1 on NK cells, monocytes, and DCs. Immune exhaustion contributes to dysfunction in lymphocytes and NK cells. Similarly, Lee et al. (30, 31) and Gao et al. (31) reported increased expression of immune receptors (IRs) on NK cells and CTLs in patients with sHLH.

Immune exhaustion in CD8^+^ T cells and NK cells leads to an impaired IFN-γ production and degranulation function, as well as a reduced cytotoxic activity against target cells. Blackburn et al. demonstrated that PD-1^high^CD8^+^ T cells showed less IFN-γ production and exhibited higher granzyme B expression compared with PD-1^low^CD8^+^ T cells in a chronic infection mouse model (25). Kamada et al. (32) reported that PD-1^+^ Tregs are less suppressive against the proliferation of CD8^+^ T cells than PD-1^−^ Tregs, suggesting their potential inability to inhibit lymphocyte proliferation and control hyperinflammation. The co-expression of multiple IRs is correlated with greater degrees of exhaustion and a more severe functional decline (33). Kelkar et al. (24) found that the percentages of PD-1^+^ CD8^+^ T cells, TIM-3^+^ CD8^+^ T cells, and LAG-3^+^ CD8^+^ T cells were significantly elevated in patients with HLH, and the proportion of PD-1^+^TIM-3^+^ CD8^+^ T cells was also increased, consistent with our result of an increased proportion of PD-1^+^TIGIT^+^CD8^+^ T cells.

We analyzed changes in the immune cell subsets before and after PD-1 inhibitor treatment in patients with EBV-HLH. PD-1 inhibition was associated with a partial immune reconstitution, including increased frequencies of cytotoxic CD8^+^ T cells (mainly T_EM_ and T_EMRA_) and CD56^dim^CD16^+^ NK cells and reduced CD163^+^ monocytes. These shifts suggest a potential reversal of exhaustion and the promotion of NK cell maturation, consistent with reports that PD-1 blockade can enhance T-cell functionality and support NK cell maturation (34, 35). However, given the limited sample size, these findings can only be interpreted as hypothesis-generating. Large-scale prospective studies are required to confirm their clinical efficacy.

## Conclusion

Adult HLH patients demonstrate skewed lymphocyte and NK cell subsets, profound immune cell exhaustion, impaired cell differentiation, and aberrant cell activation. Elevated expression levels of inhibitory receptors across multiple immune cell populations contribute to a compromised cellular functionality. PD-1 inhibitors exhibited potential in restoring lymphocyte function and facilitating viral clearance in patients with EBV-HLH. However, given the limited sample size, these findings represent a preliminary observation. In the future, large-scale prospective studies are warranted to confirm clinical efficacy and elucidate the molecular pathways and pathophysiological mechanisms underlying the observed phenotypes.

## Data Availability

The original contributions presented in the study are included in the article/**Supplementary Material**. Further inquiries can be directed to the corresponding author.
