# Supplementary material for: Flow cytometric analysis of the immune cell subsets in adult hemophagocytic lymphohistiocytosis
Source: Front Immunol. 2025 Nov 28;16:1678233. doi: 10.3389/fimmu.2025.1678233 (PMC12698659; doi:10.3389/fimmu.2025.1678233)
Supplement: Supplementary file 1 [file DataSheet1.pdf]

Supplementary Figures

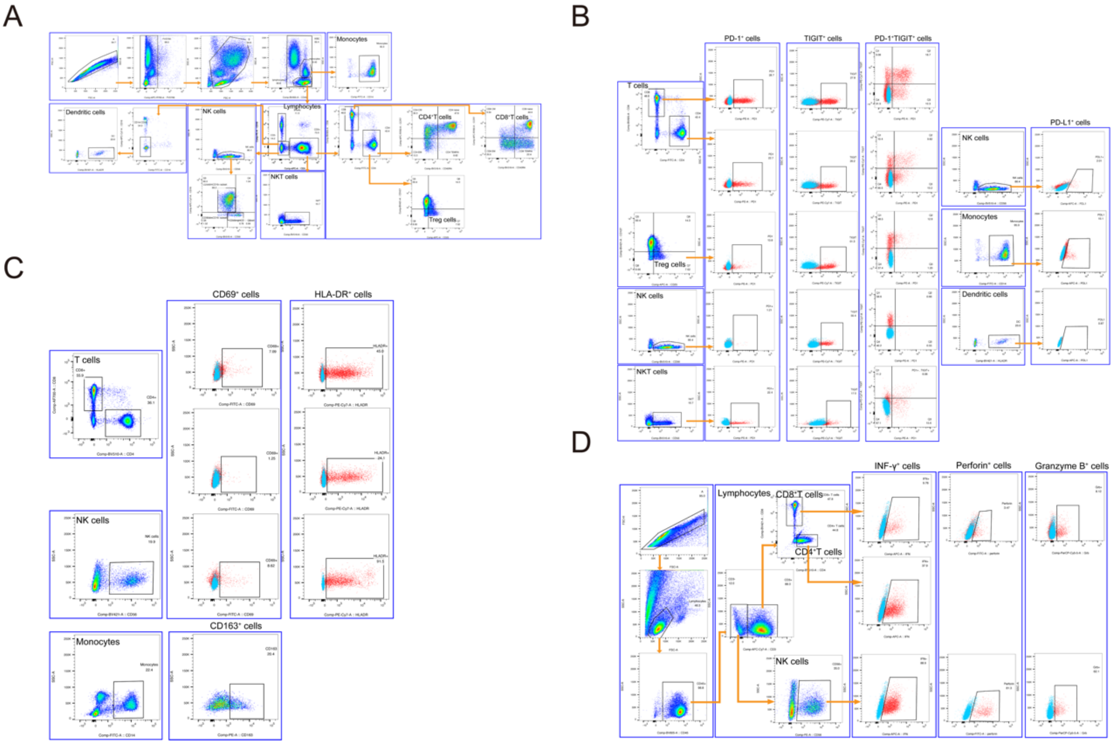

sFig. 1. Gating strategy for flow cytometric analysis of peripheral blood immune cell populations.

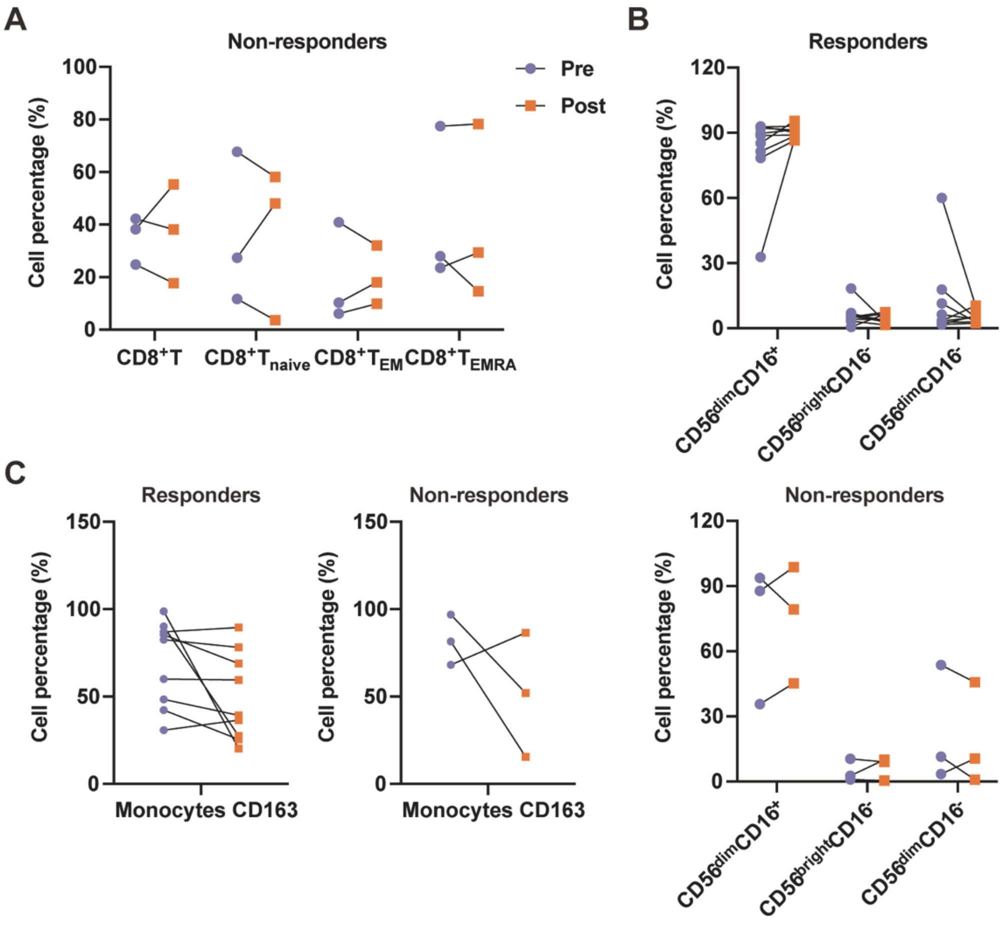

sFig. 2. Immune cell subset and functional marker changes in HLH patients based on treatment response. (A) Changes in the percentages of $CD8^+$ T cell subsets, including $CD8^+$ T_{naive}, $CD8^+$ T_{EM}, and $CD8^+$ T_{EMRA}, in non-responders before (Pre) and after (Post) treatment. (B) Changes in NK cell subsets, including $CD56^{dim}CD16^+$, $CD56^{bright}CD16^-$, and $CD56^{dim}CD16^-$, in responders and non-responders before and after treatment. (C) Percentages of $CD163^+$ monocytes in responders and non-responders before and after treatment. Data are presented as median with 95% CI.

Supplementary Tables

sTable 1. A NGS assessment panel of 42 HLH-related genes mutations

**Gene fuction**	**Genes implicated in mutations**
Familial hemophagocytic lymphohistiocytosis，FHL	PRF1、UNC13D、STX11、STXBP2
Immunodeficiency syndrome associated with HLH	RAB27A、LYST、AP3β1
X-linked lymphoproliferative disease，XLP	SH2D1A、XIAP、NLRC4、CDC42
Epstein-Barr virus-driven hemophagocytic lymphohistiocytosis	MAGT1、ITK、CD27、CD70、CTPS1、RASGRP1
Others	ADA、AP3D1、BLOC1S6、BTK、CARD11、CORO1A、DOCK2、DOCK8、FAS、IL2RA、IL2RG、MVK、MYO5A、NCKAP1L、NLRP3、NLRP12、PIK3CD、PLCG2、PNP、RAG1、RAG2、RECQL4、SLC7A7、STIM1、WAS

sTable 2. Peripheral blood immune cell subsets in HLH patients and controls

Cell subsets	HLH (n = 75)	Control (n = 10)	*P* value
Lymphocytes (%, range)			
T cell	78.70 (2.41-96.90)	73.00 (51.50-81.50)	0.202
B cell	5.72 (0.04-38.60)	11.65 (8.47-17.30)	0.097
NK cell	6.48 (0.41-73.32)	13.61 (6.20-31.22)	**0.016**
NKT cell	3.31 (0.31-38.97)	8.58 (1.01-14.71)	**0.025**
White blood cells (%, range)			
Monocyte	3.48 (0.10-29.29)	4.61 (2.70-8.84)	0.152
Dendritic cell	0.33 (0.02-12.74)	0.19 (0.09-0.55)	0.241
T cells (%, range)			
$CD4^+$	54.43 (9.67-86.10)	62.72(27.74-85.68)	0.364
$CD8^+$	39.30 (7.93-82.25)	29.64(11.02-65.89)	0.447
Treg	1.66 (0.04-8.52)	3.22 (1.14-4.60)	**0.011**
CD4/CD8	1.41 (0.12-10.82)	2.06 (0.42-7.77)	0.384
$CD4^+$ T cells (%, range)			
T_{naive}	23.17 (0-82.10)	33.70 (20.30-41.00)	0.229
T_{CM}	14.03 (0.65-60.50)	29.05 (17.70-41.90)	**0.017**
T_{EM}	45.99 (2.10-94.10)	30.70 (18.20-50.60)	0.167
T_{EMRA}	2.58 (0.14-71.53)	2.58 (1.64-10.00)	0.629
$CD8^+$ T cells (%, range)			
T_{naive}	32.84 (0.03-87.72)	18.75 (11.50-51.10)	0.210
T_{CM}	2.56 (0.00-39.53)	3.57 (0.63-19.20)	0.437
T_{EM}	29.29 (2.17-92.04)	36.35 (10.70-56.70)	0.749
T_{EMRA}	19.73 (2.83-77.44)	31.90 (20.00-75.70)	**0.006**
NK cells (%, range)			
$CD56^{dim}CD16^+$	86.00 (6.74-98.20)	98.04 (92.10-98.99)	**0.000**
$CD56^{bright}CD16^-$	4.88 (0.32-83.30)	1.16 (0.42-3.39)	**0.001**
$CD56^{dim}CD16^-$	7.69 (0.25-63.07)	0.85 (0.35-4.51)	**0.000**

NK natural killer, Treg regulatory T-cell, T_{naive} naïve T cell, T_{CM} central memory T cell, T_{EM} effector memory T cell, T_{EMRA} terminal effector memory T cell

sTable 3. The intracellular cytokine secretion function of lymphocytes in HLH patients and controls

Cell subsets	HLH (n=75)	Control (n=10)	*P* value
$CD4^+$ T cells (%, range)			
IFN-γ	18.10 (1.51-87.39)	17.00 (11.46-29.70)	0.781
$CD8^+$ T cells (%, range)			
IFN-γ	23.39 (1.73-80.50)	60.09 (14.10-80.00)	**0.008**
Granzyme B	11.98 (0.18-96.20)	2.41 (0.44-10.58)	**0.000**
Perforin	5.62 (0.06-42.90)	2.76 (0.67-28.63)	0.416
NK cells (%, range)			
IFN-γ	58.40 (12.60-99.30)	85.20 (70.90-98.26)	**0.000**
Granzyme B	36.28 (3.14-99.50)	3.43 (0.56-20.27)	**0.000**
Perforin	59.50 (8.16-95.50)	35.89 (27.06-86.05)	0.336

IFN-γ interferon gamma

sTable 4. The expression of CD107a and cytotoxic of NK cells in HLH patients and controls

Cell subsets	HLH (n=75)	Control (n=10)	*P* value
NK cells (%, range)			
CD107a (before)	9.30 (2.20-56.60)	5.44 (0.97-10.47)	**0.020**
CD107a (after)	10.29 (1.34-44.00)	11.24 (3.59-37.15)	0.611
CD107a ratio (after/before)	1.02 (0.31-2.57)	2.74 (1.24-4.71)	**0.000**
K562 cell (%, range)			
7-AAD	5.08 (0.45 -49.32)	11.10 (6.00-19.40)	**0.001**
